# Deep learning of heart-sound signals for efficient prediction of obstructive coronary artery disease

**DOI:** 10.1016/j.heliyon.2023.e23354

**Published:** 2023-12-08

**Authors:** Aikeliyaer Ainiwaer, Wen Qing Hou, Quan Qi, Kaisaierjiang Kadier, Lian Qin, Rena Rehemuding, Ming Mei, Duolao Wang, Xiang Ma, Jian Guo Dai, Yi Tong Ma

**Affiliations:** a Department of Cardiology, First Afliated Hospital of Xinjiang Medical University, State Key Laboratory of Pathogenesis, Prevention and Treatment of High Incidence Diseases in Central Asia, Urumqi, Xinjiang, 830000, China; b School of Information Network Security, Xinjiang University of Political Science and Law, Tumushuke, Xinjiang, 843802, China; c College of Information Science and Technology, Shihezi University, Shihezi, Xinjiang, 832003, China; d Emergency Department, the First Affiliated Hospital of Shihezi University, Shihezi, Xinjiang, 832003, China; e Department of Clinical Sciences, Liverpool School of Tropical Medicine, Pembroke Place, Liverpool, L3 5QU, UK

**Keywords:** Deep learning, Artificial intelligence, Obstructive coronary artery disease, Heart sound signal, Electronic stethoscope

## Abstract

**Background:**

Due to the limitations of current methods for detecting obstructive coronary artery disease (CAD), many individuals are mistakenly or unnecessarily referred for coronary angiography (CAG).

**Objectives:**

Our goal is to create a comprehensive database of heart sounds in CAD and develop accurate deep learning algorithms to efficiently detect obstructive CAD based on heart sound signals. This will enable effective screening before undergoing CAG.

**Methods:**

We included 320 subjects suspected of CAD who underwent CAG. We employed advanced filtering techniques and state-of-the-art deep learning models (VGG-16, 1D CNN, and ResNet18) to analyze the heart sound signals and identify obstructive CAD (defined as at least one ≥50 % stenosis). To assess the performance of our models, we prospectively recruited an additional 80 subjects for testing.

**Results:**

In the test set, VGG-16 exhibited the highest performance with an area under the ROC curve (AUC) of 0.834 (95 % CI, 0.736–0.930), while ResNet-18 and CNN-7 achieved AUCs of only 0.755 (95 % CI, 0.614–0.819) and 0.652 (95 % CI, 0.554–0.770) respectively. VGG-16 demonstrated a sensitivity of 80.4 % and specificity of 86.2 % in the test set. The combined diagnostic model of VGG and DF scores achieved an AUC of 0.915 (95 % CI: 0.855–0.974), and the AUC for VGG combined with PTP scores was 0.908 (95 % CI: 0.845–0.971). The sensitivity and specificity of VGG-16 exceeded 0.85 in patients with coronary artery occlusion and those with 3 vascular lesions.

**Conclusions:**

Our deep learning model, based on heart sounds, offers a non-invasive and efficient screening method for obstructive CAD. It is expected to significantly reduce the number of unnecessary referrals for downstream screening.

## 1 Introduction

The prevalence of obstructive coronary artery disease (CAD) is significant, with a projected increase of 18 % in the adult population by 2030 [1], making it twice as common as myocardial infarction [2]. Unlike acute coronary syndromes, obstructive CAD develops slowly and may present with mild symptoms, leading to frequent misdiagnoses and/or missed diagnoses [3,4], resulting in inefficient use of healthcare resources. Currently, the diagnosis of obstructive CAD relies on pretest probability (PTP), followed by additional tests such as coronary arteriography (CAG) [5,6]. As such, a straightforward and accurate screening method for obstructive CAD is urgently needed.

The concept of analysing heart sounds using spectral analysis was first proposed in 1955 [7]. Previous research has demonstrated that coronary stenosis leads to the production of high-frequency sounds caused by turbulent blood flow in partially blocked arteries [8]. This turbulence affects the surrounding coronary artery tissue, resulting in audio frequency vibrations in the adjacent myocardial wall [8]. As a result, the signal generated by coronary artery obstruction is not simply turbulence but rather a complex composite signal. This signal is characterised by weak signals amid strong background noise, typically falling within the frequency range of approximately 20–800 Hz [9]. In the early 1980s, Semmlow et al. [10], first attempted to develop a method for detecting turbulence-related sounds produced by partially occluded coronary arteries. Since then, researchers have made significant efforts in this field. Based on these studies, there is a need for a deep learning model that can directly analyze the raw audio of heart sounds to predict obstructive CAD.

The objective of this study, therefore, was to develop and validate deep learning algorithms for the detection of obstructive CAD based on heart-sound signals with the ultimate goal of enhancing screening efficiency.

## 2 Methods

### 2.1 Study design

**Fig. 1:**
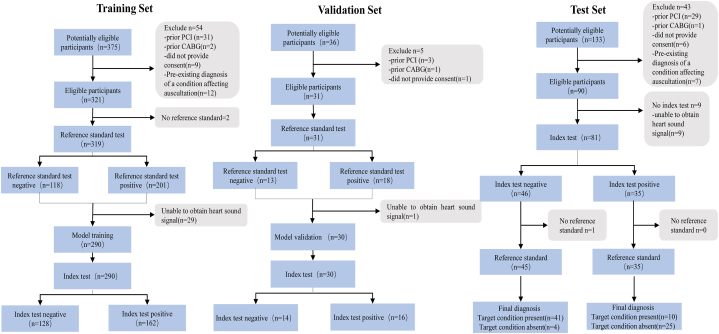
Study flowchart We obtained heart sound recordings in advance. These recordings are used to develop the DL models, and a proportion of it is used for validation. The separate testing set is used to assess the final performance of the different diagnostic models. CABG, coronary artery bypass grafting; PCI, percutaneous coronary intervention.

This study was conducted in two main phases: algorithm development; and assessment of diagnostic accuracy. In the first phase, eligible patients were enrolled and randomly divided into training and validation sets at a 10:1 ratio to develop the algorithm. Various deep learning model frameworks were used and strategies, such as normalisation, data augmentation, low-frequency denoising, and fusion for model training. In the second phase, eligible patients were enrolled in a separate test set based on specific inclusion and exclusion criteria, as depicted in Fig. 1. The primary focus during this phase was to evaluate and compare the performance of the different models.

### 2.2 Participants

This study was conducted at the First Affiliated Hospital of Xinjiang Medical University (Ürümqi, China). Patients with chronic precordial pain, assessed by outpatient physicians as potential obstructive CAD, were enrolled between April 1 and November 1, 2021. Individuals who underwent previous percutaneous coronary intervention or coronary artery bypass grafting, and those unwilling or unable to cooperate with auscultation were excluded (for details regarding controlling confounding factors, refer to the Supplementary material).

### 2.3 Data collection

Informed consent was obtained from all patients and their families. Before medical intervention, patients were asked to assume a supine position in the ward or outpatient clinic with their chest exposed and hands on their sides. Using an electronic stethoscope (Littmann Electronic Stethoscope, Model 3200, 3 M, St Paul, MN, USA) in the mixed mode, a 30 s audio recording of heart sounds was performed in each of the nine parts of the precordial region [11] (the auscultation position is described in detail in the Supplementary material) [8,10,12].

### 2.4 Research process

The reference standard for diagnosing or ruling out obstructive CAD was CAG, which assesses the degree of coronary stenosis (≥1 lesion occluding ≥50 % of the diameter of the coronary artery). Two cardiologists, who were blinded to the study design, independently reviewed the CAG images of each patient to evaluate the degree of stenosis; any disagreements were resolved through discussions with a third reviewer. CAG and obstructive CAD diagnostic criteria strictly adhered to published guidelines [13]. In cases in which patients declined CAG or had contraindications, coronary computed tomography angiography (cCTA) was used as an alternative method.

### 2.5 Data pre-processing

The heart-sound signals were processed at a sampling rate of 4000 Hz, and the mean duration of each heart sound was 25 s (approximately 30 s of heart sounds were collected, with the first and last 2.5 s removed). The collected heart sounds were converted into a matrix format for storage. Heart sounds captured at the nine locations were integrated in parallel, simulating different channels of an image, and served as the input layer of the neural network. Additional details are reported in the Supplementary Materials.

### 2.6 Data labeling

The enrolled patients were divided into two groups based on the presence or absence of obstructive CAD. Subgroups of patients with obstructive CAD were further analyzed and categorised according to sex, age, body mass index (BMI), risk factors, total number of lesions, proportion of stenoses associated with the most severe lesion, and location of the most severe lesion. The categorisations are as follows:

Sex: Classified as male or female.

BMI: Classified as > 25 kg/m^2^ and ≤25 kg/m^2^.

Age: Categorised into two groups, ≥60 and <60 years.

Risk factors: Grouped based on the presence of >3 of the following risk factors: age >40 years, male sex, diabetes mellitus, hypertension, hyperlipidaemia, BMI ≥25 kg/m^2^, and current or ex-smoker.

Number of lesions: Categorised as one-, two-, or three-vessel lesions (lesions in the left main stem were defined as two-vessel lesions).

Proportion of stenosis: Divided into the following groups based on the proportion of stenosis associated with the narrowest and most severe lesion: <50 %; 50 % ≤ lesion <70 %; 70 % ≤ lesion <100 %; and 100 % vessel occlusion.

Location of the most severe lesion: Categorised into anterior descending, circumflex, and right coronary artery groups based on the location of the most severe lesion.

### 2.7 Model design

The system used in this study was Ubuntu (Pearson Education, 2015) with Python version 3.8.3, and TensorFlow version 2.6.0. Jupyter Notebook version 6.0.3 was used as the translator.

Diagnosis of obstructive CAD using heart sounds was performed as an exploratory study with a limited sample size. Hence [14], 1D-VGG, ResNet, and convolutional neural network (CNN) networks were used for this purpose (see the Supplementary Materials for detailed steps).

Once the model architecture was finalised, the model was trained using data from 290 cases (2610 audio samples) and validated using data from 30 cases (270 audio samples). Different layers of the VGG model were tested and found that VGG-16 exhibited the best robustness in diagnosing obstructive CAD. To address the potential overfitting issues with a small training set, batch normalisation was incorporated after each convolutional layer and various model training strategies were implemented, including data augmentation, low-frequency denoising, and fusion. For a comparative analysis, a seven-layer 1D CNN was constructed and simulated a 1D ResNet18 network with residual blocks based on the ResNet18 architecture (a schematic of the Neural Network Architecture is presented in Supplemental Fig. 8).

Furthermore, three additional obstructive CAD detection models were established for performance comparison. A logistic regression model was fitted using 42 baseline variables (Supplementary materials).

Three combined diagnostic models were constructed based on heart sounds and clinical variables. The first model, VGG-16 + DF, used the Diamond-Forrester (DF) score and VGG-16 prediction scores as independent variables and the presence of obstructive CAD as the dependent variable. Similarly, the VGG-16 + PTP diagnostic model was built using the VGG-16 and PTP scores recommended by the European Society of Cardiology [13]. Finally, the VGG + LR model combined the proposed algorithm with a logistic regression model.

### 2.8 Statistical analysis

Normally distributed measures are expressed as mean ± standard deviation, and independent-samples *t*-tests were performed for intergroup comparisons. Skewed measurement data are expressed as median (interquartile range), and intergroup comparisons were performed using the Mann–Whitney *U* test. Categorical data are expressed as constituent ratios and were compared using either the chi-squared test or Fisher's exact test, as appropriate.

To assess the performance of the algorithm, various measures were calculated, including sensitivity, specificity, area under the receiver operating characteristic (ROC) curve (AUC), positive predictive value (PPV), negative predictive value (NPV), and diagnostic accuracy. A radiologist served as the comparator. The algorithm was evaluated at operating points selected from the ROC curve, specifically chosen for the maximum sum of sensitivity and specificity. Exact 95 % confidence intervals (CIs) were calculated for all diagnostic performance measures.

The Wilcoxon–Mann–Whitney test was used to compare AUCs of the different models. Additionally, prespecified subgroup analyses were performed based on age, sex, angina symptoms, risk factors, and degree of CAD. All statistical comparisons were two-sided, and differences with p < 0.05 were considered to be statistically significant. Statistical analyses were performed using SPSS version 26 (IBM Corporation, Armonk, NY, USA).

## 3 Results

### 3.1 Study population

In the training set, 198 (68 %) patients had obstructive CAD, of whom 163 were male (82 %); additionally, 92 (32 %) patients had no obstructive CAD, of whom 42 were male (46 %). In the validation set, 20 (67 %) patients had obstructive CAD, 15 of whom were male (75 %); 10 (33 %) subjects had no obstructive CAD, of whom 8 were male (80 %). The baseline characteristics of patients in the training and validation sets are summarised in Table 1 and Supplementary Table 1.

**Table 1:** Summary of participants characteristics for training, validation, and test sets.

Characteristics	Training set（n = 290）	Validation set（n = 30）	*P*^a^-value	Test set（n = 80）	*P*^b^-value
**Age**（Years）	58.75 ± 11.38	62.57 ± 9.895	0.08	58.39 ± 10.41	0.80
**Male**	206（71.00）	22（73.30）	0.79	39（48.80）	＜0.05
**Education**			0.61		0.10
**Less than high school**	105（36.20）	13（43.30）		20（36.30）	
**Highschool**	92（31.70）	7（23.30）		25（31.30）	
**University or above**	93（32.10）	10（33.30）		26（32.50）	
**Smoking**	88（30.30）	12（40.00）	0.28	24（30.00）	0.95
**Drinking**	41（14.10）	4（13.30）	1.00	21（26.30）	＜0.05
**BMI**（kg/㎡）	26（24.00,28.00）	25（24.00,28.00）	0.54	25 (22.00,28.00)	＜0.05
**Previous history**					
**HBP**	120（41.40）	14（46.70）	0.58	36（45.00）	0.56
**Diabetes**	67（23.10）	9（30.00）	1.00	15（18.80）	0.41
**Hyperlipidemia**	3（1.00）	1（3.30）	0.33	1（1.30）	1.00
**Cerebrovascular disease**	4（1.40）	2（6.70）	0.19	7（8.80）	＜0.05
**Family history**					
**CAD**	34（11.70）	6（20.00）	0.31	17（21.30）	＜0.05
**Hypertension**	27（9.30）	4（13.30）	0.70	12（15.00）	0.14
**Diabetes**	11（3.80）	1（3.30）	0.40	3（3.80）	1.00
**Cerebrovascular disease**	5（1.70）	1（3.30）	0.45	3（3.80）	0.50

Data presented as mean ± standard deviation, median and interquartile ranges or n (%). No data were missing in Table 1. BMI, body mass index; CAD, coronary artery disease; HBP, high blood pressure; pa value was obtained by comparison of the training and validation groups. pb value was obtained by comparison of the training and test groups.

In the test set, 51 (64 %) patients had obstructive CAD and 29 (36 %) did not. Significant coronary stenosis was confirmed by CAG or cCTA in 31 males (79.5 %) and 20 (48.8 %) females. Eight subjects underwent cCTA instead of CAG due to contraindications or refusal to diagnose or rule out obstructive CAD. Obstructive CAD was defined as coronary lumen stenosis ≥50 % in diameter in 26.3 % (n = 21), 16.3 % (n = 13), and 21.3 % (n = 17) of the patients with lesions involving one, two, and three vessels, respectively. Complete occlusion of a single vessel was observed in 10 patients, whereas complete occlusion of two vessels was observed in four patients. Of the participants, 36 % (n = 29) did not exhibit any significant coronary stenosis. Additionally, seven patients exhibited no significant coronary stenosis but had coronary myocardial bridging, and one experienced spontaneous coronary artery dissection (Table 1 and Supplementary Table 1).

A comparison of the baseline characteristics between the training and test sets revealed significant differences in sex, BMI, and laboratory test results (all p < 0.05).

### 3.2 Model performance

**Fig. 2:**
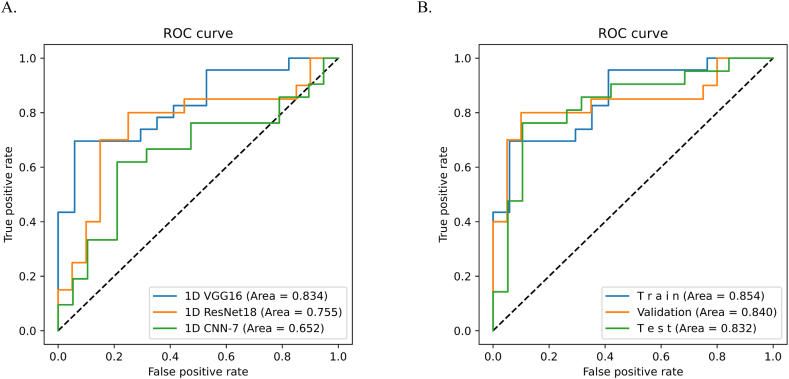
Algorithm performance for detecting obstructive CAD of different machine learning models. **A** display the ROC curves of the three models in the test group (n = 80); **B** display the ROC curve of VGG16 in training group, validation group and test group; AUC, area under the receiver operating characteristic curve (95 % CI; obstructive CAD), stable coronary artery disease; VGG, Visual Geometry Group; CNN, Convolutional Neural Networks.

Three deep-learning diagnostic models were trained: VGG-16, ResNet-18, and CNN-7. The performances of these models in the confusion matrix are reported in Supplemental Fig. 1. The ROC curves for the test set are presented in Fig. 2A. The AUC for VGG-16 was 0.834 (95 % CI 0.736–0.930), with an accuracy of 0.825 (95 % CI 0.720–0.898). In contrast, ResNet-18 and CNN-7 had lower AUC values (0.755 [95 % CI 0.614–0.819] and 0.652 [95 % CI 0.554–0.770], respectively), and accuracies of 0.725 (95 % CI 0.612–0.816) and 0.663 (95 % CI 0.5472–0.7621), respectively. When comparing the three models across the six dimensions (Table 2), VGG-16 exhibited a sensitivity of 0.804 (95 % CI 0.669–0.902), specificity of 0.862 (95 % CI 0.683–0.961), PPV of 0.911 (95 % CI 0.803–0.963), and NPV of 0.714 (95 % CI 0.585–0.816) in the test set, indicating superior performance compared with the other two models. ROC curves for the training, validation, and test sets for the VGG-16 model are presented in Fig. 2B.

**Table 2:** Performance of different models for detecting obstructive CAD in test set.

Model	AUC	SEN	SPE	PPV	NPV	ACC	F1-score	*P*-value
VGG16	0.834 (0.736, 0.930)	0.804 (0.669, 0.902)	0.862 (0.683, 0.961)	0.911 (0.803, 0.963)	0.714 (0.585, 0.816)	0.825 (0.720, 0.898)	0.854	Ref
ResNet18	0.755 (0.614, 0.819)	0.725 (0.583, 0.841)	0.724 (0.528, 0.873)	0.822 (0.715, 0.895)	0.600 (0.477, 0.712)	0.725 (0.612, 0.8161)	0.771	0.072
CNN7	0.652 (0.554, 0.770）	0.647 (0.501, 0.776)	0.724 (0.492, 0.847)	0.786 (0.673, 0.867)	0.526 (0.416, 0.634)	0.663 (0.547, 0.762)	0.71	0.009
PTP	0.761 (0.648, 0.873)	0.746 (0.652, 0.829)	0.667 (0.568, 0.759)	0.863 (0.773, 0.919)	0.483 (0.380, 0.582)	0.725 (0.619, 0.811)	0.800	0.327
DF	0.771 (0.675, 0.885)	0.807 (0.761, 0.956)	0.783 (0.457, 0.821)	0.902 (0.820, 0.948)	0.621 (0.517, 0.714)	0.8 (0.693, 0.878)	0.852	0.418
LR	0.686 (0.573, 0.784)	0.805 (0.706, 0.871)	0.538 (0.438, 0.639)	0.647 (0.547, 0.741)	0.724 (0.620, 0.803)	0.675 (0.560, 0.773)	0.717	0.032
VGG + PTP	0.908 (0.855, 0.974)	0.830 (0.739, 0.895)	0.741 (0.641, 0.820)	0.863 (0.773, 0.919)	0.69 (0.589–0.777)	0.8 (0.700, 0.873)	0.846	0.003
VGG + DF	0.915 (0.845, 0.971)	0.846 (0.762, 0.911)	0.75 (0.652, 0.829)	0.863 (0.773, 0.919)	0.724 (0.620–0.803)	0.813 (0.707, 0.888)	0.854	0.004
VGG + LR	0.883 (0.792, 0.944)	0.911 (0.832, 0.955)	0.714 (0.609, 0.794)	0.804 (0.706, 0.871)	0.862 (0.773–0.919)	0.825 (0.727, 0.893)	0.854	0.028

Delong test *P*-value <0.05 (VGG16 as reference). All values are mean values. Area under the curve was based on receiver operating characteristic analysis. PPV, Positive Predictive Value; NPV, Negative Predictive Value. SEN, sensitivity. SPE, Specificity. ACC, Accuracy. DF，Diamond-Forrester score; PTP，pre-test probability.

**Fig. 3:**
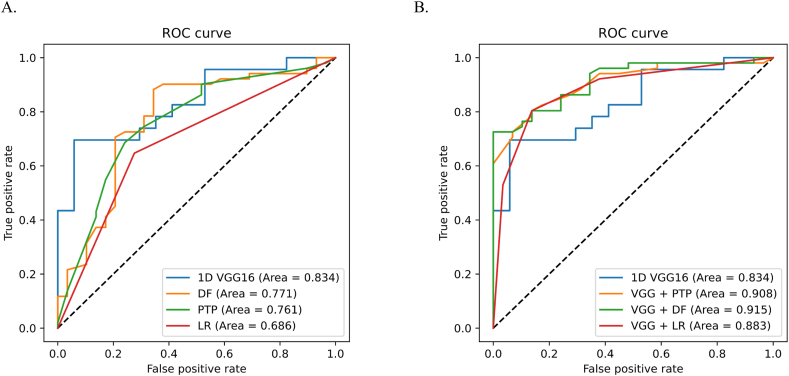
Algorithm performance for detecting obstructive CAD of clinical models and combined diagnostic models in test set (n = 80). **A** display the AUC curves for algorithm and pre-test probability scores (PTP score and DF score), logistic regression models; **B** display the AUC curves of different combined diagnostic models.

In the test set, the AUCs for the DF and PTP scores recommended by the clinical guidelines for diagnosing obstructive CAD were 0.771 (95 % CI 0.675–0.885) and 0.761 (95 % CI 0.648–0.873), respectively. The sensitivity of the DF score was 0.807 (95 % CI 0.761–0.956), while that of the PTP score was 0.746 (95 % CI 0.652–0.829). However, the specificity was lower for both scores, with values of 0.783 (95 % CI 0.457–0.821) and 0.667 (95 % CI 0.568–0.759), respectively (Fig. 3A, Table 2). The algorithm outperformed logistic regression models based on 42 baseline variables, exhibiting a greater AUC (0.833) compared with 0.686 (p = 0.032).

In the test set, three subjects were identified by cCTA as having CAD, whereas five subjects were classified as not having CAD. The VGG model correctly classified all negative subjects and two positive subjects, with only one positive subject misclassified (Supplemental Table 2).

The combined diagnostic models constructed using either the VGG-16 and DF models or the VGG-16 and PTP models outperformed the VGG-16 model alone in the test set. The AUCs were 0.908 (95 % CI 0.845–0.971) and 0.915 (95 % CI 0.855–0.974), with accuracies of 0.800 (95 % CI 0.700–0.873) and 0.813 (95 % CI 0.707–0.888), respectively (p = 0.003 and p = 0.004) (Fig. 3B, Table 2). This suggests that the combined diagnostic method can be used for outpatient diagnosis, aligning with the authors’ cardiovascular outpatient consultation process, which involves a combination of history taking and physical examination. When the algorithm was tested in conjunction with a logistic regression model containing 42 variables, joint diagnosis improved the AUC to 0.883 (95 % CI 0.792–0.944), with an accuracy of 0.825 (95 % CI 0.727–0.893).

### 3.3 Subgroup analysis

**Fig. 4:**
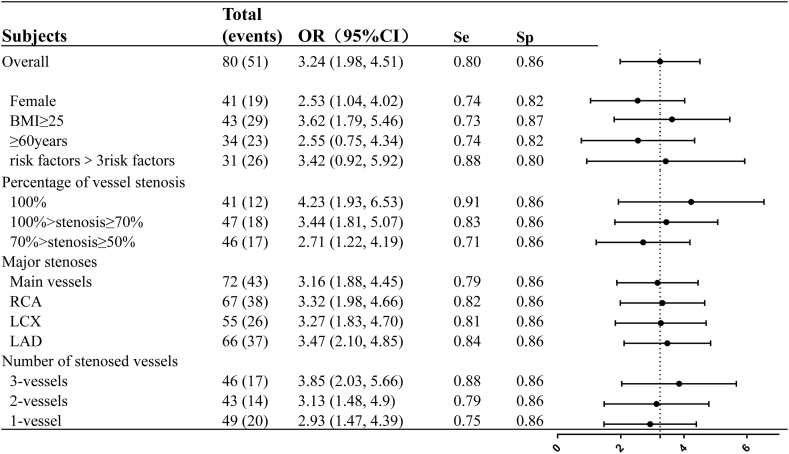
Forest plot demonstrating the subgroups of test group (n = 80). Forest plot demonstrating the subgroup results of network performance by age, gender, risk factors, percentage of vessel stenosis, major stenoses, and number of stenosed vessels. OR values refer to the correlation of each subgroup in the test set with the VGG model based on heart sound prediction CAD. The correlation of each factor with the VGG algorithm for predicting CAD was analyzed on an overall basis. Risk factors were grouped based on the presence of ＞3 of the following risk factors: age >40 years, male sex, diabetes mellitus, hypertension, Hyperlipidaemia, body mass index≥25 kg/m^2^, and current or ex-smoker; LCX, left circumflex branch; LAD, left anterior descending; RCA, right coronary artery; AUC, area under the receiver operating characteristic curve; obstructive CAD, stable coronary artery disease. OR, odd ratio (X-axis is log10 scale).

Fig. 4 presents a summary of the performance of the VGG-16 model in various subgroups of the test set, as depicted by the AUC curves provided in the Supplementary Materials. The algorithm demonstrated superior performance in individuals who were overweight (BMI ≥25 kg/m^2^), <60 years of age, had a greater number of risk factors for obstructive CAD, exhibited greater severity of stenosis, and/or had a greater number of diseased vessels.

When the target population was restricted to individuals with multiple vessel lesions (Fig. 4, Supplemental Fig. 6), the AUC, sensitivity, and specificity for predicting obstructive CAD were 0.872 (95 % CI 0.616–0.918), 0.882, and 0.862, respectively. Similarly, when the target population was limited to patients with occlusive lesions, the corresponding values were 0.889 (95 % CI 0.704–0.991), 0.917, and 0.862 and, for patients with >3 risk factors, the values were 0.842 (95 % CI 0.710–0.915), 0.885, and 0.800, respectively (Supplemental Fig. 5).

The heart-sound signals collected in this study encompassed all locations of the anterior heart vessels, which aligns with finding that the diagnostic capability of the anterior descending branch was superior to that of the circumflex branch and right coronary artery (AUC 0.850 versus [vs.] 0.835 and 0.839, respectively). For individuals with coronary stenosis ranging from 50 % to 70 %, the AUC was 0.784 (95 % CI 0.635–0.932). It is important to note that, because CAG was used as the gold standard in this study, no downstream examinations, such as fractional flow reserve measurements, were performed to further ascertain the degree of ischaemia. This limitation can introduce bias into the model training process, leading to skewed results.

Subgroup analysis based on sex revealed that the AUC for females was only 0.778 (95 % CI 0.628–0.927), which did not reach the overall model performance level of 0.832. Furthermore, in the subgroup analysis with an age limit of 60 years, it was observed that the model exhibited better performance in the younger group (i.e., <60 years of age), with an AUC of 0.973 (95 % CI 0.759–0.987). In contrast, the AUC for this model was only 0.779 (95 % CI 0.608–0.950) in the group >60 years of age.

## 4 Discussion

The present study developed and evaluated three classification algorithms that used heart-sound signals from the initial medical contact, demonstrating a high level of accuracy in detecting obstructive CAD. The use of filtering and advanced DL techniques enabled efficient CAD screening. The VGG model demonstrated excellent performance in the test set. Furthermore, the combination of the VGG model with clinical scoring significantly enhanced screening efficiency. Additionally, the diagnostic efficiency of the model improved as patient condition deteriorated.

Our findings have several significant clinical implications. First, our algorithm, which is more effective than clinical risk scores, can serve as a convenient tool for assessing the PTP of obstructive CAD based solely on heart-sound signals. Second, this method could be a valuable alternative to coronary angiography, addressing its limitations, particularly in individuals who are unable to undergo this invasive procedure. Overall, this rapid, straightforward, and convenient diagnostic method could prove to be life-saving in the prehospital phase and in situations in which invasive testing is not feasible.

Previous investigators have made significant contributions to the field of heart-sound recognition in CAD, and our study builds on their work in the early stages. Farzad et al. [15] used advanced acoustic signal analysis and discovered a distinct turbulent acoustic signal during S1–S2, specific to the coronary arteries. Simon et al. [16] developed the CADScor® System (Kongens lyngby, Hovedstaden, Denmark) [17,18], which was tested in a prospective cohort and evaluated anatomically (ICA) and haemodynamically (FFr), demonstrating a 96 % NPV when tested at a single site in the precordial region under quiet conditions. Akanksha et al. [19] used a multicore learning algorithm and processed heart-sound signals into a heat map, achieving an accuracy of 89.2 % in identifying CAD in 80 male subjects in the training set.

However, our study differs from the methodologies of Simon et al. [16] and Farzad et al. [15]. We constructed a deep learning model using original heart-sound signals [14], leveraging the advantages of deep learning methods that require less human intervention and enable more comprehensive feature extraction. Additionally, we introduced a filtering technique with a cut-off frequency of 500 Hz to eliminate noise, which is another innovative aspect of our study [20].

Similar to Akanksha et al. [19], we used the same algorithm to identify CAD in a cohort of 80 individuals. However, our method is less reliant on feature extraction, because it can directly extract features from the original audio, preserve the original characteristics, and avoid the laborious task of manual feature extraction [21]. Another innovation of our algorithm is the use of tandem convolutions, which require fewer parameters than a larger convolutional kernel [14,22].

Numerous studies have focused on machine learning CNN models for cardiovascular disease prediction using heart sounds [23]. Krishnan et al. [24] trained a CNN model using heart sounds and achieved an AUC of 0.857 for identifying patients with cardiovascular diseases.

Similarly, Bozkurt et al. [25] achieved an even higher AUC (0.877) for predicting cardiovascular disease using a CNN model trained with heart sounds. Their models appeared to be more efficient than ours in terms of disease recognition accuracy. However, there are several important differences between their study and the present study. First, the parameters used to train the models differed from those used in the present study. In addition, their models were trained using a significantly larger amount of data, whereas our data specifically targeted CAD. The most crucial disparity between our data and theirs is that our heart sounds may be classified as “normal” in their models because their data does not exclude CAD patients through CAG. Furthermore, abnormalities in the heart sounds of patients with CAD primarily occur within milliseconds following the first and second heart sounds and are less frequent [26,27]. In contrast, their trained heart sounds exhibited abnormalities during the systolic and diastolic phases with a higher frequency of occurrence. These distinctions make our study considerably more challenging, although our accuracy may be lower compared with theirs.

Semmlow et al. [10] performed a comprehensive analysis of the acoustic characteristics of coronary arteries and found that when coronary artery stenosis exceeded 85 %, the frequency of sound decreased, leading to a decline in the efficiency of CAD screening. Interestingly, the VGG-16 model exhibited superior recognition capabilities for individuals with occluded coronary arteries. One possible explanation for this observation is that a murmur associated with obstructive CAD may be a mixed murmur, potentially containing features other than the turbulent sounds produced by coronary stenosis [10,15,20,28].

The present investigation had several limitations that warrant acknowledgement. First, this was a single-centre study conducted with a limited number of prospective samples (n = 80) for validation purposes. Second, subgroup analysis revealed suboptimal performance of the VGG16 model in females, but yielded better results in overweight individuals (i.e., BMI >25 kg/m^2^). Both groups were characterised by thicker subcutaneous tissues, which may have influenced the auscultation site and, consequently, affected the results. Additionally, there was a notable disparity in the male-to-female ratio between the training and test sets (p < 0.05), which could have contributed to the relatively lower performance of the model among females. Furthermore, when conducting a subgroup analysis with an age limit of 60 years, the model demonstrated poor performance in the older age group (i.e., ≥60 years). This could be attributed to the presence of underlying diseases that may affect auscultation efficiency (AUC 0.779).

It is crucial to highlight that the electronic stethoscope used in this study, specifically the Littmann Electronic Stethoscope (Model 3200, 3 M, USA), is currently not widely available, and its production has been discontinued. This limitation may have impeded the widespread dissemination of this study. However, as the costs of electronic stethoscopes decrease, new opportunities for dissemination and implementation of this study are anticipated [29].

## 5 Conclusions

In conclusion, our findings introduce a novel concept that utilizing deep learning algorithms with raw heart sound signals can aid in the detection of obstructive CAD. This innovative technique has the potential to revolutionize the screening and monitoring of this disease, particularly for individuals who have contraindications to CAG. By addressing a critical gap in the field, this technique offers a promising solution for improving CAD diagnosis and management.

## Funding

This research was supported by State Key Laboratory of Pathogenesis, Prevention and Treatment of High Incidence Diseases in Central Asia Fund (SKL-HIDCA-2021–3) and Key Research and Development Task of Xinjiang Uygur Autonomous Region Research on Key Technologies and Optimization Strategies for Individualized Precision Diagnosis and Treatment System of Cardiovascular Diseases (2022B03022-3).

## Ethical statement

The present study complied with the term of the Declaration of Helsinki and was approved by the Institutional Review Board of the First Affiliated Hospital of Xinjiang Medical University (Ethics Approval Number: K202108- 19). Participants gave informed consent to participate in the study before taking part.

## Data availability statement

Research data is confidential. Data-sharing requests are required to meet the policies of the hospital and the funder.

## CRediT authorship contribution statement

**Aikeliyaer Ainiwaer:** Data curation, Formal analysis, Methodology, Validation, Writing – original draft, Writing – review & editing. **Wen Qing Hou:** Data curation, Formal analysis, Methodology, Writing – original draft. **Quan Qi:** Formal analysis, Methodology. **Kaisaierjiang Kadier:** Investigation, Writing – review & editing. **Lian Qin:** Investigation, Methodology. **Rena Rehemuding:** Validation, Writing – original draft. **Ming Mei:** Methodology, Writing – review & editing. **Duolao Wang:** Investigation, Methodology, Writing – review & editing. **Xiang Ma:** Data curation, Methodology, Project administration, Supervision, Validation, Writing – original draft, Writing – review & editing. **Jian Guo Dai:** Conceptualization, Data curation, Methodology, Writing – original draft, Writing – review & editing. **Yi Tong Ma:** Conceptualization, Data curation, Supervision, Validation, Writing – original draft, Writing – review & editing.

## Declaration of competing interest

The authors declare that they have no known competing financial interests or personal relationships that could have appeared to influence the work reported in this paper.

## References

[1] Mozaffarian D., Benjamin E.J., Go A.S. (2016 Jan 26). American heart association statistics committee; stroke statistics subcommittee. Heart disease and stroke statistics-2016 update: a report from the American heart association. Circulation.

[2] Priori S.G., Blomström-Lundqvist C., Mazzanti A. (2015). 2015 ESC guidelines for the management of patients with ventricular arrhythmias and the prevention of sudden cardiac death: the task force for the management of patients with ventricular arrhythmias and the prevention of sudden cardiac death of the European society of Cardiology (ESC)endorsed by: association for European paediatric and congenital Cardiology (AEPC). Eur. Heart J..

[3] Patel M.R., Calhoon J.H., Dehmer G.J. (2017). ACC/AATS/AHA/ASE/ASNC/SCAI/SCCT/STS 2016 appropriate use criteria for coronary revascularization in patients with acute coronary syndromes: a report of the American college of Cardiology appropriate use criteria task force, American association for thoracic surgery, American heart association, American society of echocardiography, American society of nuclear Cardiology, society for cardiovascular angiography and interventions, society of cardiovascular computed tomography, and the society of thoracic surgeons. J. Am. Coll. Cardiol..

[4] Duffy E.Y., Cainzos-Achirica M., Michos E.D. (2020). Primary and secondary prevention of cardiovascular disease in the era of the coronavirus pandemic. Circulation.

[5] Siontis G.C., Mavridis D., Greenwood J.P. (2018). Outcomes of non-invasive diagnostic modalities for the detection of coronary artery disease: network meta-analysis of diagnostic randomised controlled trials. BMJ.

[6] Želízko M., Toušek F., Skalická H. (2014). Summary of the 2013 ESC guidelines on the management of stable coronary artery disease: prepared by the Czech Society of Cardiology. Cor Vasa.

[7] Mckusick V.A., Webb G.N., Humphries J.O. (1955). On cardiovascular sound: further observations by means of spectral phonocardiography. Circulation.

[8] Schmidt Se, Hansen J., Zimmermann H. (2011). Coronary artery disease and low frequency heart sound signatures. Comput. Cardiol..

[9] Schmidt Se, Holst-Hansen C., Hansen J. (2015). Acoustic features for the identification of coronary artery disease. IEEE Trans. Biomed. Eng..

[10] Semmlow J., Rahalkar K. (2007). Acoustic detection of coronary artery disease. Annu. Rev. Biomed. Eng..

[11] Oliynik V. (2015). 2015 IEEE 35th International Conference on Electronics and Nanotechnology (ELNANO).

[12] Akay M., Akay Y.M., Gauthier D. (2009 Feb). Dynamics of diastolic sounds caused by partially occluded coronary arteries. IEEE Trans. Biomed. Eng..

[13] Knuuti J. (2020). 2019 ESC Guidelines for the diagnosis and management of chronic coronary syndromes the Task Force for the diagnosis and management of chronic coronary syndromes of the European Society of Cardiology (ESC). Russ. J. Cardiol..

[14] Simonyan K., Zisserman A. (2014). Very Deep Convolutional Networks for Large-Scale Image Recognition. Comput. Sci..

[15] Azimpour F., Caldwell E., Tawfik P., Duval S., Wilson R.F. (2016). Audible coronary artery stenosis. Am. J. Med..

[16] Winther S., Nissen L., Schmidt S.E. (2018). Diagnostic performance of an acoustic-based system for coronary artery disease risk stratification. Heart.

[17] Bjerking L.H., Hansen K.W., Biering-Sørensen T. (2021). Cost-effectiveness of adding a non-invasive acoustic rule-out test in the evaluation of patients with symptoms suggestive of coronary artery disease: rationale and design of the prospective, randomised, controlled, parallel-group multicenter FILTER-SCAD trial. BMJ Open.

[18] Winther S., Schmidt S.E., Holm N.R. (2016). Diagnosing coronary artery disease by sound analysis from coronary stenosis induced turbulent blood flow: diagnostic performance in patients with stable angina pectoris. Int. J. Cardiovasc. Imaging.

[19] Pathak A., Mandana K., Saha G. (2022). Ensembled transfer learning and multiple kernel learning for phonocardiogram based atherosclerotic coronary artery disease detection. IEEE J. Biomed. Health Inform..

[20] Pathak A., Samanta P., Mandana K., Saha G. (2020). An improved method to detect coronary artery disease using phonocardiogram signals in noisy environment. Appl. Acoust..

[21] Wu J., Liu X., Zhang X. (2018). Master clinical medical knowledge at certificated-doctor-level with deep learning model. Nat. Commun..

[22] Ioffe S., Szegedy C. (2015). http://arxiv.org/abs/1502.03167.

[23] Ainiwaer A., Kadier K., Qin L., Rehemuding R., Ma X., Ma Y.T. (2023). Audiological diagnosis of valvular and congenital heart diseases in the era of artificial intelligence. Rev. Cardiovasc. Med..

[24] Krishnan P.T., Balasubramanian P., Umapathy S. (2020). Automated heart sound classification system from unsegmented phonocardiogram (PCG) using deep neural network. Phys. Eng. Sci. Med..

[25] Bozkurt B., Germanakis I., Stylianou Y. (2018). A study of time-frequency features for CNN-based automatic heart sound classification for pathology detection. Comput. Biol. Med..

[26] Larsen B.S., Winther S., Nissen L. (2021). Spectral analysis of heart sounds associated with coronary artery disease. Physiol. Meas..

[27] Zhou X., Guo X., Zheng Y., Zhao Y. (2023). Detection of coronary heart disease based on MFCC characteristics of heart sound. Appl. Acoust..

[28] Makaryus A.N., Makaryus J.N., Figgatt A. (2013). Utility of an advanced digital electronic stethoscope in the diagnosis of coronary artery disease compared with coronary computed tomographic angiography. Am. J. Cardiol..

[29] Jain A., Sahu R., Jain A., Gaumnitz T., Sethi P., Lodha R. (2021). Development and validation of a low-cost electronic stethoscope: DIY digital stethoscope. BMJ Innov..

