# Supplementary material for: Deep learning of heart-sound signals for efficient prediction of obstructive coronary artery disease
Source: Heliyon. 2023 Dec 8;10(1):e23354. doi: 10.1016/j.heliyon.2023.e23354 (PMC10758826; doi:10.1016/j.heliyon.2023.e23354)
Supplement: Multimedia component 1 [file mmc1.docx]

Supplementary Material

Deep learning of heart sound signals for efficient prediction of obstructive coronary artery disease

**Aikeliyaer Ainiwaer^1†^, Wen Qing Hou^2†^, Quan Qi^2^, , Kaisaierjiang Kadier^1^, Lian Qin^1^, Rena Rehemuding^1^, Ming Mei^2^, Duolao Wang^3^, Xiang Ma^1*^,Jian Guo Dai^2*^, Yi Tong Ma^1*^.**

*** Correspondence:** Xiang Ma: maxiangxj@ yeah. net; Jian Guo Dai: [djg_inf@shzu.edu.cn](mailto:djg_inf@shzu.edu.cn); Yi Tong Ma, myt_xj@ sina.com.

**Contents**

**Supplementary Methods**

1. Protocol for heart sound signals collection

2. Logistic regression model

3. Model design

**Supplementary Figures**

Supplemental Figure 1, Supplemental Figure 2, Supplemental Figure 3, Supplemental Figure 4, Supplemental Figure 5, Supplemental Figure 6, Supplemental Figure 7, Supplemental Figure 8.

**Supplementary Tables**

Supplemental Table 1, Supplemental Table 2

**Supplementary Methods**

**1. Protocol for heart sound signals collection**

According to the projection of the heart structure on the heart surface and the alignment of the coronary artery on the heart surface, nine sites in the precordial region were selected as heart sound collection sites:

1) the intersection of the midline of the sternum and the third rib space;

2) the intersection of the midline of the sternum and the fourth rib space;

3) the intersection of the midline of the sternum and the fifth rib space;

4) the intersection of the left edge of the sternum and the third rib space;

5) the intersection of the left edge of the sternum and the fourth rib space;

6) the intersection of the left edge of the sternum and the fifth rib space;

7) the clavicle 3 cm inward from the intersection of the midline and the third rib space;

8) 2 cm inward from the intersection of the midline and the fourth rib space;

9) approximately 1.5 cm inward from the intersection of the midline and the fifth rib space.

Audio Evaluation：

The following cases are excluded: a). Any patient with less than 9 heartbeat audios or less than 20s per audio; b). Noise in audio that can be captured by the human ear(For example, talking voice, coughing voice, etc.) and c). Contraindicated for CAG or cCTA.

The following conditions will be used for training: a). Patient has a previous diagnosis of cardiac disease, no murmur or a small murmur in the assessment of the auscultator; b). Anterior cardiac discomfort with a previous diagnosis of rhythmical heart disease

**2.** **Multivariable Logistic Regression Results for the Clinical Model to Predict Coronary Artery Disease**

Age（Years）,gender, Education, Symptom, Smoking, Drinking, BMI（kg/㎡）, DBP（mmHg）, SBP（mmHg）, Pulse rate（bpm）, Previous history(include: HBP, Diabetes, Hyperlipidemia, Cerebrovascular disease), Family history(CAD, HBP, Diabetes, Cerebrovascular disease, TnI（ng/L）, CK-MB（U/L）, MYO (μg/L), BNP (pg/Ml), WBC（×10^9^ /L）, HGB（g/L）, K^+^ (mmol/L), Na^+^(mmol/L), Glu (mmol/L), TG (mmol/L), TC (mmol/L), HDL (mmol/L), LDL (mmol/L), CRP (mg/L), IL-6 (pg/ml), PCT (pg/ml), D-dimer(mg/L), HCY (μmol/L), HbAlc(mmol/mol), Cl-(mmol/L), EF (%), Reduced wall motion.

**3. Model design**

Traditionally, disease-related clinical variables/features were manually selected to construct models, and the data sources are highly subjective in nature. Our study aims to develop an intelligent method that can automatically diagnose sCAD by heart sounds. The model can make appropriate CAD classification decisions based on the input original heart sound signals. CAD diagnosis with the help of heart sounds is a very exploratory study with a limited sample size. Therefore, we chose the 1D-VGG networks, which is similar to the 2D-VGG network and has the same characteristics of local connectivity and weight sharing. Among them, the local connectivity uses spatial topology to establish non-fully connected spatial relationships between neighboring layers to reduce the number of parameters that need to be trained, whereas weight sharing is used to avoid over-fitting of the algorithm.

We simulated VGG-16 to construct a 16-layer 1D convolutional neural network (CNN), with convolutional kernel set to 3. A 1-D max pooling layer was added after the 2nd, 4th, 7th, 10th, and 13th convolutional layers to retain the main features while reducing parameters and computation, so as to prevent over-fitting and improve the generalization of the model. All the convolutional layers were activated by the Rectified Linear Unit (ReLU) function. Finally, after 13 layers of convolution and corresponding pooling operations, the data were flattened into a 1D vector using Flatten (), followed by three fully-connected layers, with Softmax as the final layer of the classifier to output the classification results.

After the architecture of the model was completed, we used the data of 290 cases (2610 audios) to train the model and those of 30 cases (270 audios) for validation. We chose VGG-16 as our model. In order to make the neural network better fit the heart sound data and identify the optimal parameters, we used the binary cross-entropy loss as the loss function and applied the Adam optimization, with the learning rate, beta-1, beta-2, and clip being 0.001, 0.9, 0.999 and 1, respectively. To avoid training bias, the model was established using an equal number of negative and positive samples as the input batches in each extended training set.

After each epoch, the network was tested using the internal validation dataset, during which the network hyperparameters were also adjusted during this process, and the network with the lowest binary cross entropy loss value was selected once the loss value on the validation set stopped decreasing for 5 epochs. When the model performed well in the internal validation set, we saved the model. We selected a 7-layer 1D-CNN, a 16-layer 1D-CNN, and an 18-layer 1D-CNN consisting of residual blocks and tested the model performance change as the number of network layers increased. The VGG-16 diagnostic model was constructed after the test showed that the 16-layer 1D-CNN was more suitable for heart sound.

To compare the experimental results, we constructed a 7-layer 1D CNN and simulated ResNet18 to construct a 1D ResNet18 network with residual blocks. For the 7-layer 1D CNN, one 1D Maxpooling layer was added after the 2nd, 4th, and 7th convolutional layers, with Rule as the activation function in each layer. Finally, Softmax was used as the classifier to output the classification results. In 1D ResNet18, similar to the conventional ResNet18 network, 17 convolutional layers and one fully-connected layer were used, and the whole network was divided into four large Resnet blocks. Each Resnet block included two residual blocks, and the final outputs were binary classification results.

**Supplementary Figures**

Supplemental Figure 1 confusion matrix of VGG-16


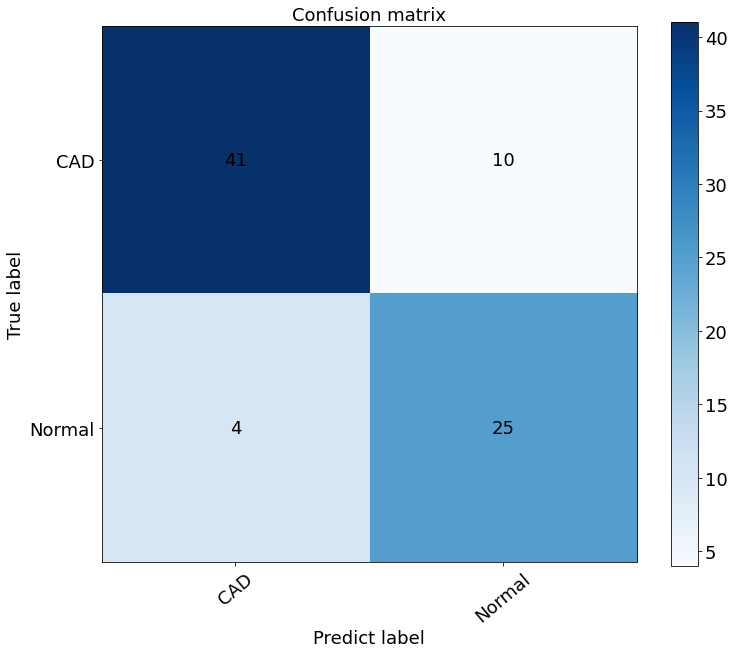


Supplemental Figure 1 The accuracy of the model in classifying heart sounds correctly when judged against labels provided by the technique professionals.

Supplemental Figure 2 confusion matrix of ResNet18

**
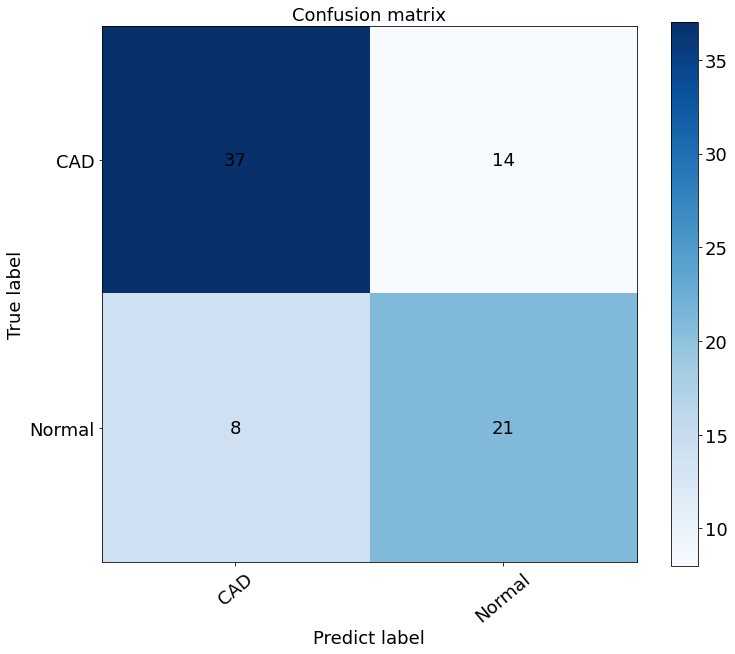
**

Supplemental Figure 2 The accuracy of the model in classifying heart sounds correctly when judged against labels provided by the technique professionals.

Supplemental Figure 3 confusion matrix of CNN-7


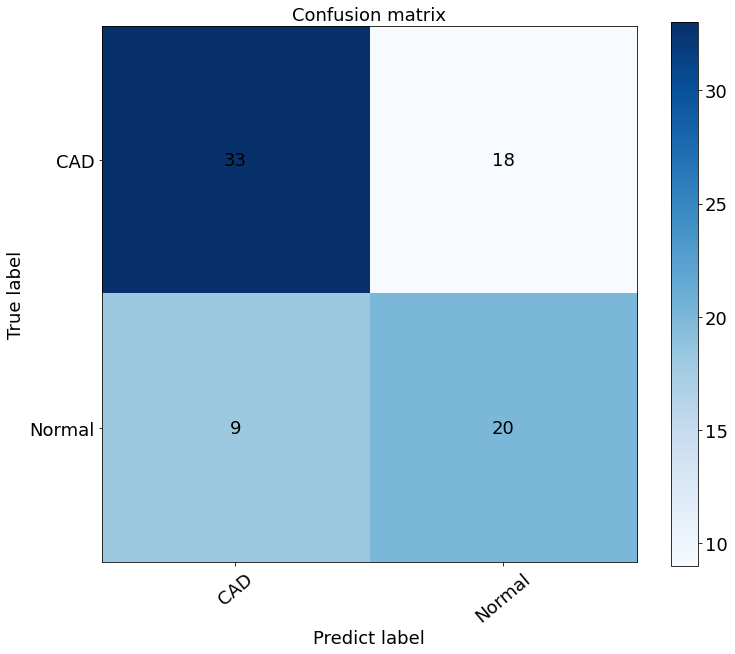


Supplemental Figure 3 The accuracy of the model in classifying heart sounds correctly when judged against labels provided by the technique professionals.

Supplemental Figure 4.


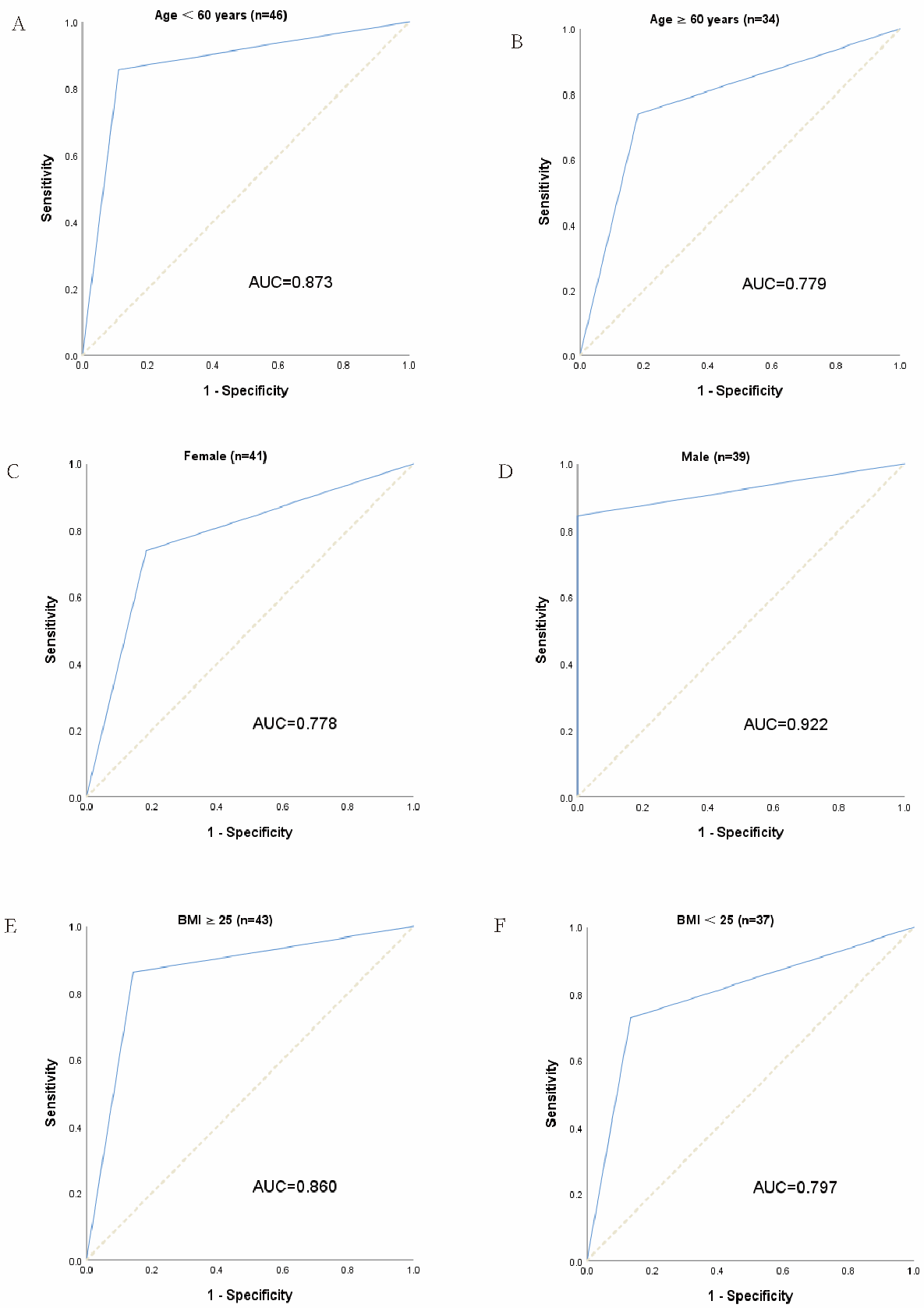


Algorithm performance in subgroups of test group. AUC, area under the receiver operating characteristic curve;

Supplemental Figure 5.


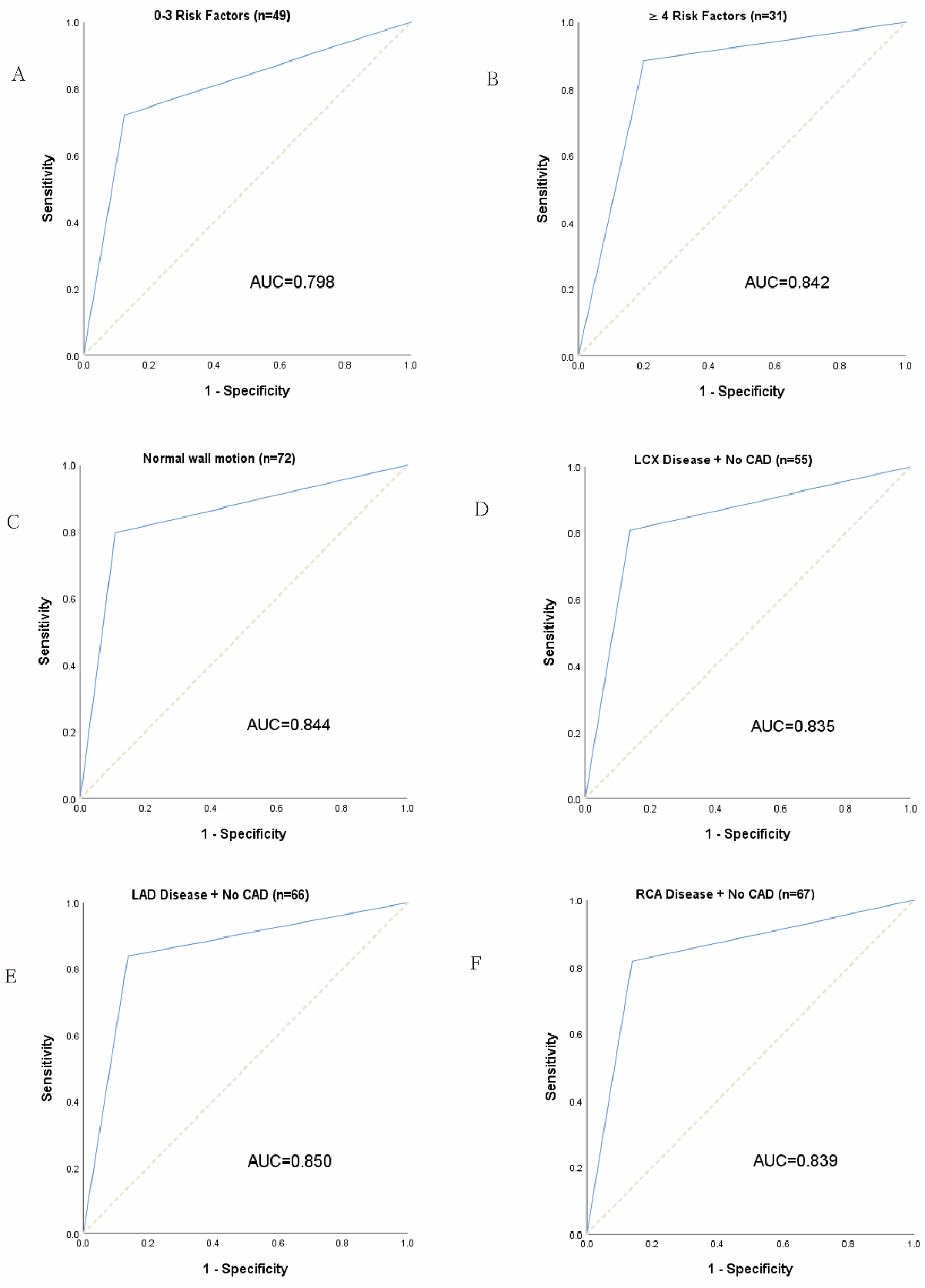


Algorithm performance in subgroups of test group. AUC, area under the receiver operating characteristic curve;

Supplemental Figure 6.


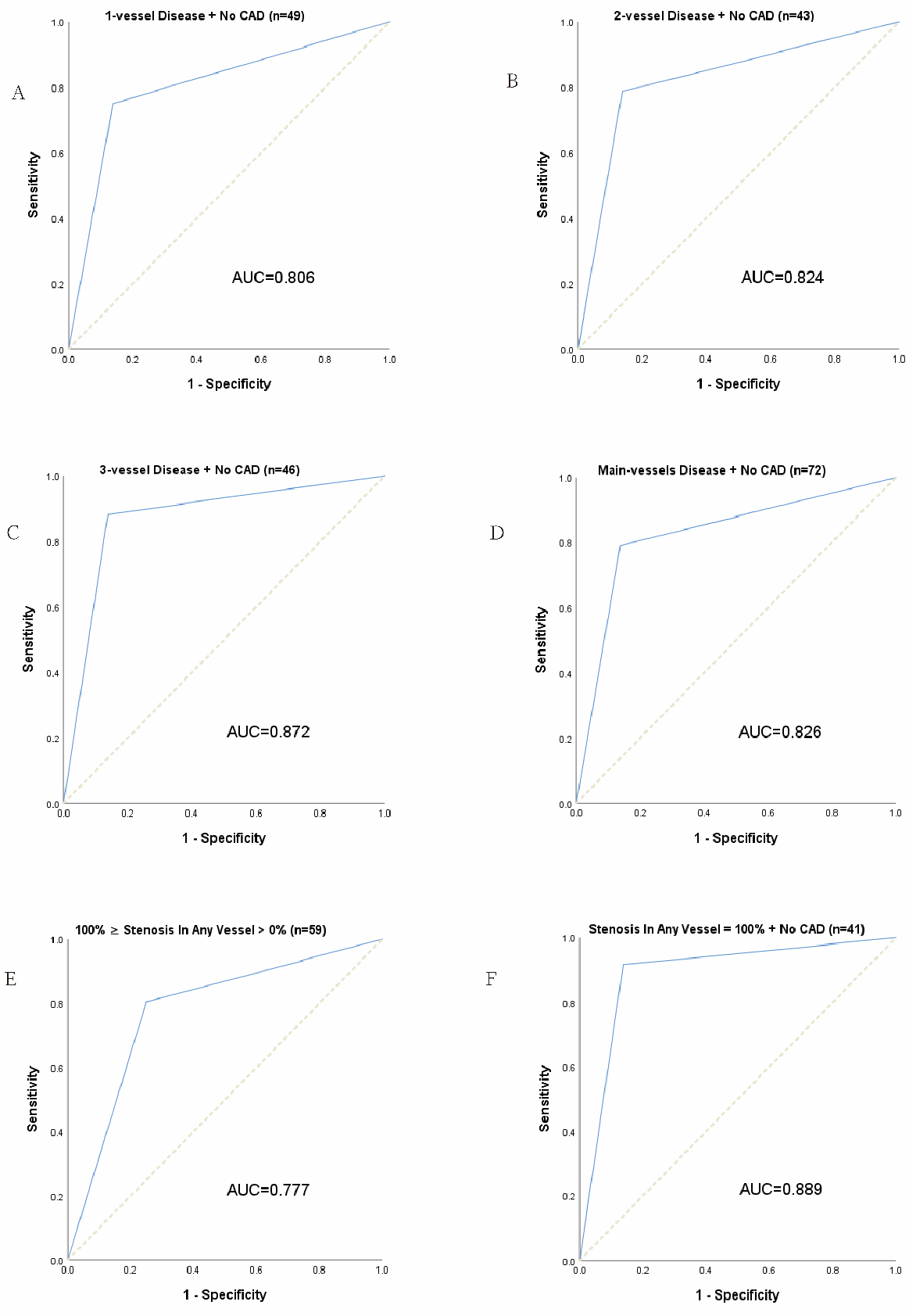


Algorithm performance in subgroups of test group. AUC, area under the receiver operating characteristic curve;

Supplemental Figure 7.


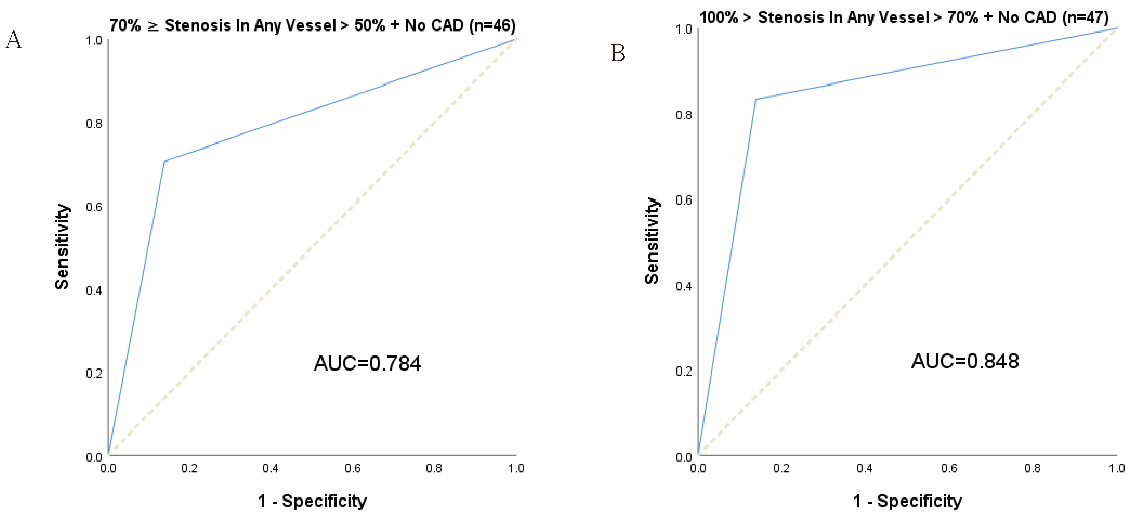


Algorithm performance in subgroups of test group. AUC, area under the receiver operating characteristic curve;

Supplemental Figure 8. Schematic of the Neural Network Architecture.


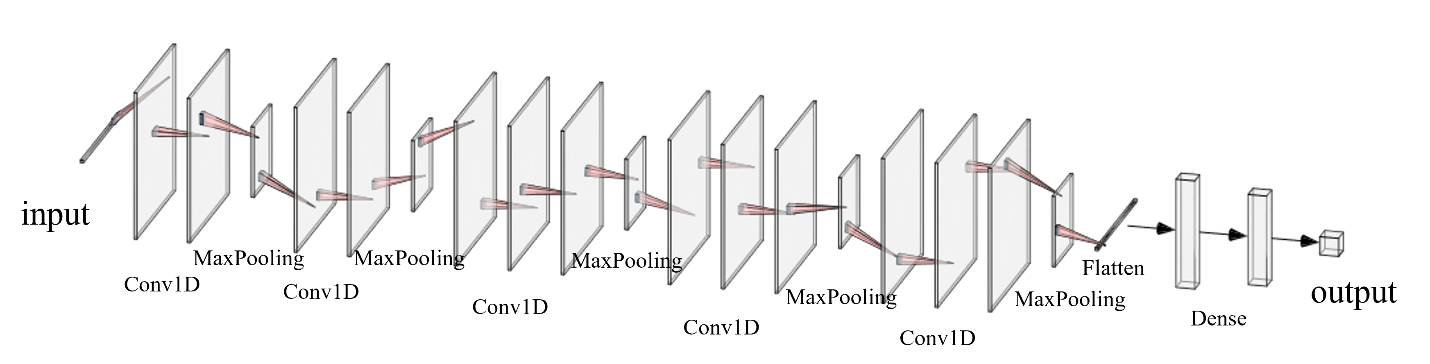


|  |
| --- |

As shown in the figure8, the 16-layer 1DCNN is divided into five convolutional blocks, in which the first two convolutional blocks contain two convolutional layers (Conv1D) and a maximum pooling layer (Maxpooling), and the last three convolutional blocks contain three convolutional layers and a maximum pooling layer, followed by a Flatten layer to "flatten" the input. The latter three convolutional blocks contain three convolutional layers and a maximum pooling layer, followed by a Flatten layer to flatten the input, i.e., to one-dimensionalize the multidimensional input, filter the data from the convolutional layer to the fully connected layer, and finally classify it by softmax.

| \| **Characteristics** \| **Training set** \| **Validation set** \| ***P*^a^-value** \| **Test set** \| ***P*^b^-value** \| \| --- \| --- \| --- \| --- \| --- \| --- \| \| **Symptom** \|  \|  \| 0.93 \|  \| 0.11 \| \| **Typical angina** \| 277（95.50） \| 28（93.30） \|  \| 72（90.00） \|  \| \| **Atypical angina** \| 13（4.50） \| 2（6.70） \|  \| 8（10.00） \|  \| \| **DBP**（mmHg） \| 77（69.00,84.00） \| 75（68.00,80.00） \| 0.41 \| 77.5(69.50,86.50) \| 0.47 \| \| **SBP**（mmHg） \| 126（117.00,138.00） \| 129（120.00,140.00） \| 0.26 \| 126.5(115.50,141.50) \| 0.70 \| \| **Pulse rate**（bpm） \| 77（72.00,84.00） \| 74.5（70.00,80.00） \| 0.32 \| 76(72.00,82.00) \| 0.78 \| \| **Days of hospitalization** \| 4（4.00,6.00） \| 4（4.00,5.00） \| 0.93 \| 4(3,4) \| ＜0.05 \| \| **Medication** \|  \|  \|  \|  \|  \| \| **Antiplatelet drugs** \| 87（30.00） \| 10（33.30） \| 0.71 \| 21（26.30） \| 0.51 \| \| **Statins** \| 78（26.90） \| 8（26.70） \| 0.98 \| 21（26.30） \| 0.91 \| \| **ACEI** \| 20(6.90) \| 2(6.70) \| 1.00 \| 1(1.30) \| 0.10 \| \| **ARB** \| 31（10.70) \| 4(13.30) \| 0.89 \| 16(20.00) \| ＜0.05 \| |
| --- | --- | --- | --- | --- | --- | --- | --- | --- | --- | --- | --- | --- | --- | --- | --- | --- | --- | --- | --- | --- | --- | --- | --- | --- | --- | --- | --- | --- | --- | --- | --- | --- | --- | --- | --- | --- | --- | --- | --- | --- | --- | --- | --- | --- | --- | --- | --- | --- | --- | --- | --- | --- | --- | --- | --- | --- | --- | --- | --- | --- | --- | --- | --- | --- | --- | --- | --- | --- | --- | --- | --- | --- | --- | --- | --- | --- | --- | --- |

**Supplemental Table 1 continued**

| **Characteristics** | **Training set** | **Validation set** | ***P*^a^-value** | **Test set** | ***P*^b^-value** |
| --- | --- | --- | --- | --- | --- |
| **Medication** |  |  |  |  |  |
| **β-blocker** | 58(20.00) | 6(20.00) | 1.00 | 10(12.50) | 0.13 |
| **Nitrates** | 10(3.40) | 3(10.00) | 0.21 | 3(3.80) | 1.00 |
| **Antidiabetic drug** | 59(20.30) | 8(26.70) | 0.42 | 16(20.00) | 0.946 |
| **CCB** | 62(21.40) | 10(33.30) | 0.14 | 14(17.50) | 0.45 |
| **TnI**（ng/L） | 0.012（0.012,0.017） | 0.012（0.012,0.015） | 0.86 | 0.012(0.012,0.013) | 0.22 |
| **CK-MB**（U/L） | 0.795（0.52,1.24) | 0.695(0.47,1.05) | 0.15 | 0.645(0.42,0.95) | ＜0.05 |
| **MYO** (μg/L) | 29.155(21.18,40.96) | 29.89(26.18,38.89) | 0.42 | 25.77(21.09,33.80) | 0.05 |
| **BNP** (pg/Ml) | 97.25(45.00,233.00) | 91.2(38.10,208.00) | 0.62 | 58.5(34.05,154.00) | ＜0.05 |
| **WBC**（×109 /L） | 6.81(5.77,8.05) | 6.34(5.24,6.93) | 0.06 | 6.450(5.56,7.66) | 0.33 |
| **HGB**（g/L） | 143(133.00,153.00) | 139(130.00,151.00) | 0.20 | 140(132,150) | 0.17 |
| **K^+^** (mmol/L) | 3.78(3.57,4.00) | 3.675(3.59,3.98) | 0.31 | 3.78(3.62,4.05) | 0.97 |

**Supplemental Table 1 continued**

| **Characteristics** | **Training set** | **Validation set** | ***P*^a^-value** | **Test set** | ***P*^b^-value** |
| --- | --- | --- | --- | --- | --- |
| **Na^+^**(mmol/L) | 141.67(140.00,143.11) | 141.155(139.12,143.08) | 0.56 | 141.895(139.63,143.74) | 0.52 |
| **Glu** (mmol/L) | 6.175(5.20,8.08) | 6.455(5.78,8.10) | 0.42 | 5.665(5.10,7.22) | 0.11 |
| **TG** (mmol/L) | 1.75(1.24,2.37) | 1.48(1.04,2.04) | 0.05 | 1.705(1.06,2.54) | 0.42 |
| **TC** (mmol/L) | 3.985(3.24,4.83) | 4.075(3.02,5.02) | 0.89 | 4.175(3.52,4.93) | 0.08 |
| **HDL** (mmol/L) | 0.93(0.76,1.14) | 1.07(0.81,1.24) | 0.07 | 1.09(0.96,1.27) | ＜0.05 |
| **LDL** (mmol/L) | 2.295(1.71,2.96) | 2.235(1.92,3.19) | 0.64 | 2.545(2.06,3.12) | 0.07 |
| **CRP** (mg/L) | 5.8(5.00,8.12) | 6.8(5.50,7.90) | 0.09 | 6.4(5.70,7.65) | ＜0.05 |
| **IL-6** (pg/ml) | 2.56(1.56,4.24) | 2.32(1.80,2.96) | 0.35 | 2.595(1.87,3.53) | 0.87 |
| **PCT** (pg/ml) | 0.03(0.02,0.03) | 0.03(0.02,0.05) | 0.14 | 0.03(0.02,0.04) | ＜0.05 |
| **D-dimer**(mg/L) | 85.5(58.00,152.00) | 97.5(58.00,126.00) | 0.92 | 74.0(48.00,127.5) | 0.14 |
| **HCY** (μmol/L) | 11.865(10.05,13.67) | 11.075(9.04,13.15) | 0.20 | 10.81(9.85,12.64) | ＜0.05 |
| **HbAlc**(mmol/mol) | 6.0(5.60,6.60) | 6.1(5.50,6.80) | 0.91 | 5.75(5.45,6.30) | ＜0.05 |
| **Cl^-^**(mmol/L) | 105.61±2.91 | 105.51±2.84 | 0.86 | 106.22±3.07 | 0.10 |
| **EF** (%) | 62.4(60.09,63.69) | 62.68(60.92,64.43) | 0.26 | 62.975(62.14,64.20) | ＜0.05 |
| **Reduced wall motion** | 76(26.20) | 3(10.00) | 0.05 | 8(10.00) | ＜0.05 |

**Supplemental Table 1 continued**

Supplemental Table 1.Data presented as mean ± standard deviation, median and interquartile ranges or n (%). No data were missing in Table 1. ACEI, angiotensin-converting enzyme inhibitor; BMI, body mass index; CCB, calcium channel blocker; CAD, coronary artery disease; HBP, high blood pressure；SBP, systolic pressure; DBP, diastolic blood pressure; PCT, Procalcitonin; Glu, Glucose; ***P***^a^ value was obtained by comparison of the training and validation groups. ***P***^b^ value was obtained by comparison of the training and test group.

**Supplemental Table 2. Basic information about the 8 subjects who received cCTA in the test set**

| **Heart sound number** | **VGG predicted results** | **cCTA** | **Basic Characteristics** |
| --- | --- | --- | --- |
| 327 | Negetive | Negetive | 50-year-old female, in menstruation, patient considered for cCTA, not for CAG at this time |
| 343 | Positive | Positive | 62-year-old female who refused to have CAG and requested conservative treatment. |
| 360 | Positive | Positive | 66-year-old male with pulmonary infection status, recommended to treat respiratory disease first and perform CAG after the condition improves. |
| 377 | Negetive | Negetive | 53-year-old male with unexplained fecal occult blood, CAG not recommended. |
| 386 | Positive | Positive | 49-year-old male refused CAG and requested conservative treatment. |
| 392 | Negetive | Negetive | 68 year old male refused CAG and requested cCTA. |
| 396 | Negetive | Negetive | 57 year old male, CAG not considered due to allergy to aspirin. |
| 398 | Negetive | Positive | 51-year-old female, refused to undergo CAG, cCTA was positive, but the patient still refused to undergo further tests or treatment. Patient has a history of prior pancreatic surgery and prediction error was considered related to this. |
